# Why empathy has a beneficial impact on others in medicine: unifying theories

**DOI:** 10.3389/fnbeh.2014.00457

**Published:** 2015-01-14

**Authors:** Jean Decety, Aikaterini Fotopoulou

**Affiliations:** ^1^Department of Psychology, University of ChicagoChicago, IL, USA; ^2^Department of Psychiatry and Behavioral Neuroscience, University of Chicago MedicineChicago, IL, USA; ^3^Research Department of Clinical, Educational and Health Psychology, University College LondonLondon, UK

**Keywords:** empathy, free energy principle, health, medicine, neuroscience, predictability, social support, social baseline theory

## Abstract

The past decades have seen an explosion of studies on empathy in various academic domains including affective neuroscience, psychology, medicine, and economics. However, the volumes of research have almost exclusively focused on its evolutionary origins, development, and neurobiological bases, as well as how the experience of empathy is modulated by social context and interpersonal relationships. In the present paper, we examine a much less attended side of empathy: why it has a positive impact on others? After specifying what the construct of empathy encompasses, we brieﬂy review the various effects of empathy on health outcomes in the domain of medicine. We then propose two non-mutually exclusive mechanistic explanations that contribute to explain the positive effects of physician empathy on patients. (1) The social baseline theory (SBT), building on social support research, proposes that the presence of other people helps individuals to conserve metabolically costly somatic and neural resources through the social regulation of emotion. (2) The free energy principle (FEP) postulates that the brain optimizes a (free energy) bound on surprise or its complement value to respond to environmental changes adaptively. These conceptualizations can be combined to provide a unifying integrative account of the benefits of physicians’ empathetic attitude on their patients and how it plays a role in healing beyond the mere effect of the therapeutic alliance.

## Introduction

It is widely agreed upon that empathy is a good thing, and that it should be the basis of attitudes towards patient care, or should at least play an important role in the doctor-patient relationship alongside deductive logic, physical examinations, and treatment. More and more often, medical education underscores the importance of empathy in medicine, and a growing number of medical schools have specific educational programs and initiatives for their students (Shapiro, 2012). In parallel, during the past decades, a wealth of studies and theoretical research has examined the construct of empathy from numerous perspectives including philosophy, psychology, clinical neuroscience, and affective and social neuroscience (Farrow and Woodruff, 2007; Decety and Ickes, 2009; Coplan and Goldie, 2011; Decety, 2012; Coutinho et al., 2014). However, most of this academic research has focused on the empathizer (i.e., the person who experiences empathy), and much less effort has been directed toward proposing a mechanistic explanation of why empathy positively impacts the other (i.e., the person who receives empathy).

In this article, we examine the current knowledge in neuroscience to offer an integrative and comprehensive perspective on the neurobiological and cognitive mechanisms that underlie the positive role of empathy in medicine. The avenues by which empathy affects the well-being of the recipient may be very diverse and include both dispositional and situational factors. We begin by examining what the notion of empathy comprises. Next, we brieﬂy review the evidence supporting the positive inﬂuence of empathy on health outcomes in the context of medicine. We then propose that two theoretical frameworks, the social baseline theory (SBT) and the free energy principle (FEP), contribute in explaining the positive effects of empathy on various health outcomes, including pain-related variables.

## Empathy is about emotion and caring

A fundamental assumption of emotion theory is that emotion is an automatic orienting system that evolved to guide adaptive behavior. Emotion is also, however, a means of interpersonal communication that evokes responses from other conspecifics. Thus, emotions can be viewed both as intrapersonal and interpersonal states, and the construct of empathy incorporates both such dimensions (Decety and Skelly, 2014), and reﬂects an intersubjective induction process by which positive and negative emotions are shared, without losing sight of whose feelings belong to whom (Decety and Meyer, 2008). Empirical and theoretical work in developmental science, psychology, and affective neuroscience converge to consider empathy as a natural competency that has evolved with the mammalian brain to form and maintain social bonds, necessary for surviving, reproducing and maintaining well being, and which comprises dissociable facets (Decety and Svetlova, 2012; Decety et al., 2012). These different components include: (1) Affective sharing, the first element of empathy to appear during ontogeny. It reﬂects the capacity to become affectively aroused by the valence and intensity of others’ emotions. (2) Empathic understanding, which entails the conscious awareness of the emotional state of another person. (3) Empathic concern, which refers to the motivation to care for someone’s welfare. (4) Cognitive empathy, similar to the construct of perspective taking or theory of mind is the ability to put oneself into the mind of another individual and imagine what that person is thinking or feeling (Decety and Jackson, 2004; Goubert et al., 2009; Singer and Lamm, 2009; Derntl et al., 2010; Decety, 2011; Decety and Cowell, 2014). Given these multifaceted aspects of empathy, there is no single region in the brain that underlies this ability. Rather, the circuits involved in emotional saliency (amygdala, insula and anterior cingulate cortex), central executive network (dorsolateral prefrontal cortex, posterior parietal cortex), and caregiving (brainstem, hypothalamus, basal ganglia, and ventromedial prefrontal cortex) constitute independent and tightly coupled networks that support the experience of empathy (Decety and Cowell, 2014). Furthermore, the neural pathways involved empathy and caring are facilitated and modulated by neuroendocrine mechanisms. In particular, the neuropeptide oxytocin, which plays a general role in social interactions by reducing stress and anxiety (Anacker and Beery, 2013; Cardoso et al., 2013), and as a result enhances cognitive empathy (Rodrigues et al., 2009) and empathic concern (Shamay-Tsoory et al., 2013; Smith et al., 2014).

In medicine, empathy is generally conceptualized as a communication competence, as well as a subjective experience between an observer (the physician) and a subject (the patient or client), in which the observer, uses various sensory cues (body language, prosody, etc.), to identify and transiently experience the subject’s emotional states (Hirsch, 2007). The physician’s emotional attunement serves the cognitive goal of understanding patients’ emotions (Halpern, 2012). Additionally, empathy is generally viewed by the patient as the doctor’s ability to understand how he/she feels and thinks, as well as how the doctor expresses concern, compassion, and care for the patient’s own well-being. Both of these aspects contribute to patient satisfaction. Thus, in the medical context, all facets of empathy (affective, cognitive, and motivational) are important and can be adaptively engaged to positively inﬂuence patients’ health.

## Empathy and compassionate health care

**Figure 1:**
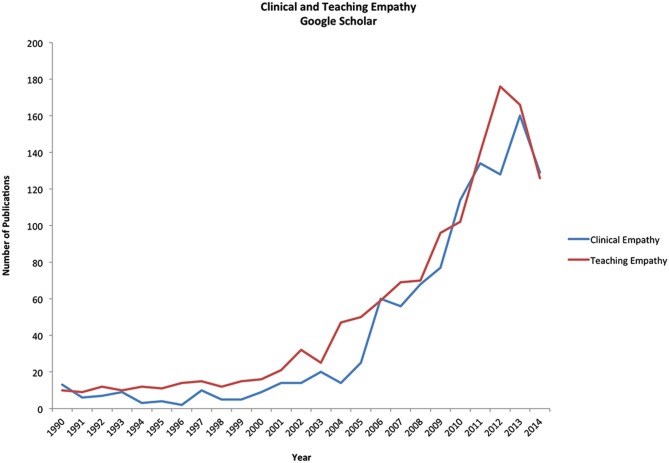
**Number of articles on the topics of clinical empathy and teaching empathy published between 1990 until 2014, from a Google Scholar search**.

In spite of the conceptual diversity that characterizes the notion of empathy, this concept is widely used in patient-centered practices and increasingly prominent in contemporary medical education (Pedersen, 2010). It is worth noting, however, that some authors argue that empathy is neither necessary nor sufficient to guarantee good medicine (Smajdor et al., 2011). While empathy has always been considered an essential component of compassionate care, in the past years, there has been a veritable tsunami of publications on the importance of empathy in patient care, how it can be improved, and how it can be taught in medical school. A Google search with “teaching empathy to medical students” gives over 1,410,000 hits (Figure 1).

There are several reasons why empathy can be valuable in medical practice, and these explain the surge of interest and enthusiasm in the concept with regards to patient-physician relationships. For instance, in the practice of psychiatry and clinical psychology, an empathetic attitude allows the therapist to gather invaluable information about the patient and contributes to building the reliable, trustworthy relationship that lies at the heart of real safety and growth in therapy. Clinical empathy is also an important element of quality health care in medicine. Physicians who attempt to understand what their patients are feeling, whether they are successful (empathic accuracy) or just genuinely communicating their concern (empathic concern), achieve a number of valuable outcomes for their patients (Mercer and Reynolds, 2002). Moreover, such communication takes place with both verbal and non-verbal means. For example, a doctor’s caring touch as opposed to a diagnostic touch is perceived as conveying clinical empathy and promotes healing (Montague et al., 2013).

More importantly for the scope of our argument, patient perception of physician’s empathy is associated with various improved health outcomes. Patients give fuller histories, disclosing more to physicians who are non-verbally attuned to them (Halpern, 2012). Empathic communication is associated with improved patient satisfaction, increased adherence to treatment, and fewer malpractice complaints, as well as increased physician health, well-being, and professional satisfaction (Riess et al., 2012; Gleichgerrcht and Decety, 2013).

Importantly, cognitive empathy can improve patient satisfaction. In a randomized study, student–clinicians who were given a perspective-taking intervention received significantly better patient satisfaction scores from standardized patients than did controls (Blatt et al., 2010). Several empirical studies reported that patients’ perceptions of their physicians’ empathy are positively related to more favorable health outcomes. One study found that diabetic patients of physicians with high empathy scores were significantly more likely to have good control of hemoglobin A1c than were patients of physicians with low empathy scores (Hojat et al., 2011). Logistic regression analyses demonstrated that physicians’ empathy had a unique contribution to the prediction of optimal clinical outcomes after controlling for physicians’ and patients’ gender and age, and patients’ health insurance.

Empathic concern has been strongly implicated in patient adherence to medical regimens, with research documenting a direct relationship between patient-perceived physician empathy and increased satisfaction with and compliance to treatment. For instance, a large scale study reported that clinician empathy and caring attitude, as perceived by patients with the common cold, significantly predicted subsequent duration and severity of illness and was associated with more robust immune system responses, as measured by levels of inﬂammatory cytokine in nasal secretions (Rakel et al., 2009). To explore the factors associated with patient enablement in general practice consultations, one study of over 3,044, showed that patients’ perceptions of the doctors’ empathy was of key importance in their enablement in general practice consultations in both high and low deprivation settings individuals (Mercer et al., 2012). In addition, maximal patient enablement was never found with low empathy.

In medicine, empathy has important implications, not only for the patient health outcomes, but also for physician success. Doctors who show empathy have less malpractice complaints and better patient satisfaction and compliance (Huntington and Kuhn, 2003). This finding is especially notable, as there are areas in the United States where the cost of malpractice insurance can be prohibitive, causing physicians to leave medicine (Huntington and Kuhn, 2003). A meta-analysis, reviewing contextual effects related to the patient-practitioner relationship in clinical populations with a physical illness, reported that while there is much inconsistency regarding the effect of emotional and cognitive care, physicians who adopt a warm, friendly, and reassuring manner are more effective than those who keep consultations formal and do not offer reassurance (Di Blasi et al., 2001). Mindfulness-based interventions that enhance attention, awareness, and communication skills, increase empathy and improve the physician’s well being and emotional stability (Krasner et al., 2009).

While it is true that most studies of the effect of the patient-clinician relationship on medical outcomes are observational in nature and therefore cannot assess causality, a recent meta-analysis of thirteen randomized controlled trials indicates that relationship factors between physician and patients hold an important potential that affects health outcomes (Kelley et al., 2014).

It is important to note that being too empathetic can be costly for the health care practitioner (Gleichgerrcht and Decety, 2012). However, research suggests that a modicum level of personal distress (or emotional sharing/attunement), is necessary for the physicians’ professional quality of life (Gleichgerrcht and Decety, 2014). Because physicians are exposed to high levels of negative emotions in stressful environments, they can indeed develop compassion fatigue and severe emotional exhaustion (Figley, 2012), which may impede the delivery of quality medical care and increase the risk of medical errors. Thus, it can be challenging for health-care professionals to find a delicate balance between over-identification with their patients and emotional detachment. Therefore, emotional regulation skills are critical for physicians to keep their emotions under control and maintain personal stability (Cheng et al., 2007; Decety, 2009; Decety et al., 2010). Psychological and neuroscience research indicate that individuals who can regulate their own affective responses to maintain an optimal level of emotional arousal have greater expressions of empathic concern for others (Decety and Meyer, 2008; Davidov et al., 2013; Ho et al., 2014; Williams et al., 2014).

**Figure 2:**
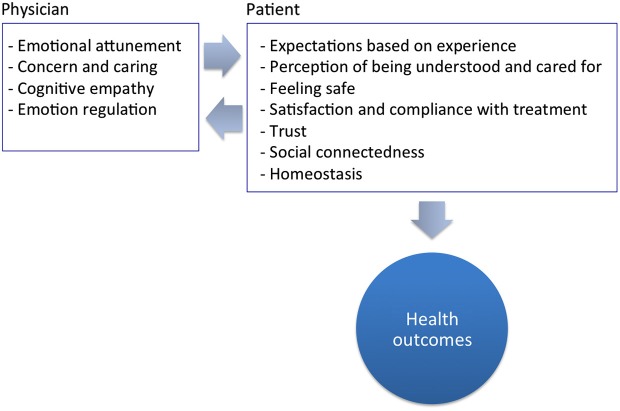
**The effect of empathy in medicine: clinical empathy results from the integration of cognitive, behavioral, and emotional factors (both in the empathiser and the receipient of empathy) and is embedded in an interaction between a physician and a patient**.

Overall, there is solid and accumulative evidence that all facets of empathy play an important role in medical practice and have an impact on both the patient and his/her physician (Figure 2). A physician’s empathy can improve the patient’s psychological and physiological adjustment to disease, contribute to healing, and can inﬂuence the overall well being of the recipient; a fact that calls for a mechanistic explanation.

## The social baseline theory

Social support is one of the most important functions of social relationships. Numerous studies indicate that it is essential for maintaining physical and mental health, and a lack of support is associated with harmful consequences (Ozbay et al., 2007). A wealth of evidence suggests that perceived availability of a significant other’s (or family or friends generally) support ameliorates stress and is associated with health and well being (Di Blasi et al., 2001; Taylor, 2007; Reblin and Uchino, 2009). Conversely, a lack of support and social isolation are important predictors of morbidity and major risk factors for psychological illness (Cacioppo and Hawkley, 2009; Cacioppo et al., 2012), as well as mortality (Reblin and Uchino, 2009). Based on both animal models and human studies, the proposed mechanisms for these “social buffering” effects include the regulation of stress-related activity in the autonomic nervous system (ANS), and hypothalamic-pituitary-adrenal (HPA) axis (Uchino et al., 1996; Hennessy et al., 2009; for a recent excellent comprenhensive review, see Hostinar et al., 2014). Social neuroscience research in humans has long investigated the neurocognitive mechanisms by which social support inﬂuences physiology and ultimately health. For instance, in a handful of recent neuroimaging studies, social support was found to reduce activity in brain regions that are typically implicated in emotional and homeostatic regulation. In particular, neuro-hemodynamic changes were detected in the anterior cingulate cortex, dorsolateral and ventrolateral prefrontal cortex and midbrain regions (Coan et al., 2006; Eisenberger et al., 2007).

The social baseline theory (SBT) proposes that organisms are adapted to social ecology—the presence of other conspecifics—more so than any physical ecology. Consequently, the social proximity to other individuals (characterized by familiarity, joint attention, shared goals, and interdependence) should be considered as the default or baseline assumption of the human brain (Beckes and Coan, 2011; Coan, 2011). The SBT is grounded in empirical studies that have found that neural pathways and hormonal stress responses associated with self-regulation of emotion are less active when social support is provided or even anticipated (Gunnar and Quevedo, 2007; Hostinar et al., 2012). The neural response to threat cues is minimized when a high-quality relational partner is on hand. Individuals whose relationships are marked by perceived mutuality and responsiveness are characterized by decreased self-regulatory effort and by reduced preparation to respond to threat cues (Coan et al., 2013). Importantly, animal research demonstrates similar physiological effects. For instance, one study found that female mice approaching a dyad member in physical pain led to less writhing from the mouse in pain (Langford et al., 2010). These positive effects of social approach were seen only when the mouse was a cage mate of the mouse in pain and not for a stranger. These results replicate previous findings reporting reduced pain sensitivity in mice when interacting with siblings, but no such analgesic effect when mice interact with a stranger (D’Amato and Pavone, 1993).

Similarly in humans, when their significant other was present, patients with fibromyalgia (a chronic widespread musculoskeletal pain, morning stiffness, insomnia, and fatigue) reported less pain sensitivity and showed diminished hemodynamic response to the tactile stimulation of a tender point compared to when the patients were alone (Montoya et al., 2004). In healthy women, holding their partner’s hand during anticipation of a shock was associated with a pervasive attenuation of activation in the neural systems supporting emotional threat response including the hypothalamus, anterior cingulate cortex, and dorsolateral prefrontal cortex (Coan et al., 2006). Finally, it appears that even a mere photograph of one’s partner is enough to reduce the perception of experimentally-induced pain and related neural responses (Master et al., 2009; Younger et al., 2010).

The SBT is a useful framework for examining individual differences in the social baselines of individuals, in particular regarding their attachment styles. Attachment is an innate biological system promoting proximity seeking between an infant and a specific attachment figure in order to increase the likelihood of survival, and empathy and attachment are interdependent (Kestenbaum et al., 1989; Mikulincer et al., 2001; Decety and Svetlova, 2012). Attachment theory offers a compelling platform for understanding an individual’s capacity to connect with others and develop supportive relationships including coping resources (Mallinckrodt, 2000). Studies have shown a mediational relationship between attachment style and empathy, and have indicated that individuals with secure attachment histories are more responsive to and caring for others (Weinfield et al., 2010). Interestingly, attachment insecurity has been associated with reduced pain thresholds and is a prognostic factor for chronic pain (Meredith et al., 2008). It has been further shown that insecure attachment may lead to increased perception of experimentally-induced pain particularly in the presence of empathic observers (Sambo et al., 2010; Hurter et al., 2014). More generally, attachment style moderates the benefits of social support, such that insecurely attached individuals report less perceived social support (Collins and Feeney, 2004) and securely attached individuals report lower state anxiety levels (Ditzen et al., 2008). Presumably, individuals that are more insecure in their relationships regard supportive others as less reliable, rendering them unable to invest wholeheartedly in their social network (Gross and Proffitt, 2013), and less likely to benefit from social support and empathy (Krahé et al., 2013).

Empirical work has begun to uncover some of the underlying neuroanatomical and neurochemical foundations of attachment-related processes and their relationship with other social behaviors. Such research has elucidated a number of neuropeptides that are clearly involved in an array of attachment-related social behaviors including opioids, vasopressin, and oxytocin. Studies in humans have demonstrated that oxytocin infusion can modulate a number of attachment-related behaviors including trust, empathic concern, and empathic accuracy (Graustella and MacLeod, 2012). Intranasal oxytocin administration has recently been shown to affect cerebral blood perfusion in critical areas in the brain circuitry involved in social cognition and emotional processing, irrespective of any concomitant cognitive, affective, or social manipulations (Paloyelis et al., 2014). Importantly, oxytocin administration selectively lessens affective reactions to threatening social stimuli (Norman et al., 2011) and differentially modulates visual attention toward social signals of positive approach (Domes et al., 2013). Moreover, it appears that individuals lacking high quality social connections show significantly decreased responses to oxytocin administration (Norman et al., 2011), a finding that may reﬂect reduced receptor sensitivity in these individuals. Research into the inﬂuence of genetic variation within the oxytocin receptor has provided converging evidence of the role that oxytocin plays in human social behavior. Polymorphisms within the oxytocin receptor have been shown to be related to affiliative behavior, dispositional empathy, theory of mind, and perceived social connectedness (Kumsta and Heinrichs, 2013). Similarly, genetic variation in the OT receptor is related to decreased neuroendocrine and autonomic reactivity to social stress, and interacts with perceived social support to dampen physiological reactivity to social-evaluative threat (Kumsta and Heinrichs, 2013).

Overall, there is a growing body of research showing that emotions provide rapid, embodied information about current resources and contextual demands, guiding decision making by modifying the subjective perception of the world (Coan and Maresh, 2014). Through the establishment and maintenance of social relationships, the resources of relational partners (parents, friends, spouses), as well as physicians, come to be viewed as resources available to the individual. Such processes optimize energy expenditures, and in the context of medicine play a critical role in buffering against stress, especially when the physician is perceived to have a compassionate and caring attitude. The SBT predicts that computational or metabolic resources devoted to attention and self-regulation are conserved through social proximity and interaction. This notion needs to be combined with the FEP, which accounts for action, perception, and salience processing on the basis of a single energy optimization principle.

## The free energy principle

The free energy principle (FEP) can explain how contextual and social factors, such as a clinician’s empathy, may affect physical and mental health outcomes. The starting point of the FEP is that biological systems minimize a free energy function of their internal states, which rely on beliefs about hidden states in their environment (Friston, 2010). The signals our sensory systems receive from the outside world are often ambiguous. Such ambiguity can have negative consequences for humans and other animals that need to use their environment to maintain their bodily homeostasis (e.g., avoid cold climates, eat safe food, etc.). According to this framework, the brain attempts to deal with the tension between a variable world and the need for homeostasis by constructing inferential hypotheses (i.e., “generative models”) of the hidden causes of sensory input it receives from the environment so as to be able to predict the changing world to a certain extent (Dayan et al., 1995). The brain further uses errors in the accuracy of such predictive representations to improve its own models. In other words, the nervous system compares its predictions with the feedback it actually receives from the physical and social environments (Friston, 2005). In order to formalize this kind of inferential self-organization, theoretical neuroscientists use Bayesian theory and terms from information theory, as well as neurocomputational schemes such as the predictive coding hypothesis (Hinton and van Camp, 1993). Sufficient coverage of the mathematical considerations of these computations is not possible within the scope of the current paper. Instead, we offer a psychological translation of this framework and particularly its application to cognitive, affective, and social neuroscience, so as to examine the effects of empathy and social support on human health and disease from the perspective of a wider framework of mental and brain functioning. In psychological terms therefore, the framework’s main proposal is that the ultimate goal of the brain is to minimize its prediction errors in perception (build better models about sensory data) and in action (sample the world to generate more sensory evidence that may fulfill its predictions). This minimization is thought to take place by recurrent feedback among hierarchical neural levels. In each level, higher levels send predictions to lower units, while such units communicate prediction errors to higher levels of the hierarchy to improve the organism’s models. Furthermore, the relative inﬂuence of predictions vs. prediction errors across several layers in this hierarchical organization is determined by the weighting (precision) of predictions vs. prediction errors at each level. Such ‘precision’ is thought to correspond to neural modulation. For example, in a hungry individual, dopaminergic action on certain synapses may enhance the weighting of any prediction errors regarding the availability of food stimuli, so that the potential presence of food acquires psychological salience for the individual, no matter how unexpected that presence may be.

Applying this large-scale theory of brain function to the understanding of the communicative and healing facets of empathy is advantageous as it can provide a mechanistic, unified account of the relation between bottom-up (e.g., neurophysiological) and top-down (e.g., psychosocial) inﬂuences on health. This approach is compatible with existing biopsychosocial models but has the advantage of offering direct links between these different factors, instead of treating them as merely additive variables. Indeed, this framework is already used to provide a neurobiological explanation of other domains of social cognition such as the neurocognitive basis of theory of mind (Brown and Brüne, 2012; Koster-Hale and Saxe, 2013), and related psychopathologies such as autism (Pellicano and Burr, 2012; Lawson et al., 2014).

Crucially, the FEP has also been used to account for the mounting experimental evidence regarding the social modulation of pain (Krahé et al., 2013). Primary signals about the homeostatic state of the organism, including nociceptive signals, are viewed as represented in the posterior insula (Craig, 2002). There they are integrated with exteroceptive information about the body and the environment. Further re-mappings within the anterior insula and the anterior cingulate cortex are thought to allow further integrations with social, motivational, and contextual information. These remappings ultimately give rise to the conscious experience of emotions and readiness for action (Craig, 2002). Moreover, the insular cortex is thought to process interoceptive stimuli on the basis of salience, determining the balance between bottom-up and top-down information about the significance of an impending noxious stimulus (Wiech et al., 2010). Pain can thus be understood as a process of perceptual inference. Predictive, top-down signals about an impending nociceptive stimulus and information about its homeostatic significance in context of other stimuli and conditions in the environment determine the conscious perception of pain. Furthermore, within the FEP framework, pain can also be seen as a parallel process of active inference. Self-protective, motivated actions are performed to change homeostatically-relevant nociceptive input and update predictions. More generally, such re-representations of signals about the inside of the body, its surface and the environment suggest possible, hierarchical neurobiological mechanisms by which top-down cognitive and social factors such as empathy can inﬂuence interoception and other signals to give rise to emotions and motivated behavior.

In this way, the FEP contributes to our understanding of how empathy may affect pain and other dimensions of physical and psychological health. When a patient seeks assistance from a physician, or when she sees a nurse approaching, both bottom-up sensory input (e.g., eye contact, warm language, gentle touch) and top-down expectancies (e.g., knowledge of therapeutic intentions based on verbal interactions and past experiences) are essential for the ensuing predictive processes of neural inference by which healing may take place. For example, in the domain of pain, social variables may affect the conscious perception of physical pain, as well as subpersonal, physiological reactions to pain (Sambo et al., 2010), by affecting the precision of impending noxious stimuli in relation to the precision of top-down predictions of pain. This balance can be affected in at least two primary ways. First, others can signal the safety or threat of the impending noxious stimulus itself and hence increase the precision of bottom-up signals regarding its salience for the organism. For example, pain tolerance can increase when a social partner re-interprets noxious, uncomfortable stimuli as neutral or positive sensations (Jackson et al., 2005) while socially sharing threatening information about the stimulus can decrease pain tolerance (Jackson et al., 2009). Second, pain and related reactions can be affected by social signals about the safety or threat of the environment in which the stimulus occurs. For example, social interactions with a clear content regarding the provision of safety or support seem to increase the perception of environmental safety and reduce the perception of experimentally-induced pain (Brown et al., 2003).

Importantly, this framework explains both the positive and negative effects of empathy, as well as the individual differences in the perception of the physician’s empathy and its effects on health. For example, it appears that while thinking of one’s partner during the experience of pain may be beneficial for nociceptive and stress perception (Younger et al., 2010). Knowing that your partner feels empathy for you during the experience of pain may lead to increased perception of pain and related behaviors (Hurter et al., 2014). It seems that such empathy is interpreted as signaling that something dangerous and salient is happening to the individual. In addition, the particular direction of these effects seems to depend on individual traits and strategies used to cope with physical or social threat. For instance, individuals with higher attachment avoidance, who generally have low trust in others and lower expectations of empathy (Hurter et al., 2014), report more pain in the presence of others than alone (Sambo et al., 2010). Also, when anxious and secure individuals perceive greater empathy from their partners, they seem to use increased displays of pain to seek emotional support. Conversely, avoidant individuals, who did not expect such a response, seem to behaviorally downplay their pain to avoid further emotional and potentially active support from their partners (Hurter et al., 2014).

Lastly, under the free energy framework, the perceptual and motivational aspects of empathy on health can be unified under the same optimization principle. Specifically, during stressful situations individuals are motivated to act in order to avoid danger. In the FEP, such motivational aspects can be conceptualized as a process of active inference, where actions are performed to change sensory signals related to homeostasis and thus reduce prediction errors. In a social context, such actions may be directed towards the environment with the goal of eliciting help from others and changing sensations via this indirect social channel.

Thus, perceiving others’ empathy may be an important way to ensure safety, while also preserving resources that may be needed during illness or bodily threat. Unfortunately, this later aspect of the effect of empathy on health has not yet received sufficient empirical attention, and hence these predictions remain untested.

## Integration of the two frameworks

The two frameworks considered here, namely the SBT, and the FEP, stem from different theoretical and empirical traditions and appear quite distinct at first sight. The SBT suggests that social relatedness is the default condition the brain expects the organism to be, while the FEP seems to describe the constitution of the human condition as relating primarily to the singular individual and its body. Nevertheless, at a closer inspection, one can see how a theoretical integration of the two perspectives is not only possible, but it may further generate some valuable insights for both frameworks, as well as for the topic at hand, namely the importance of empathy and social support for mental and physical health and disease.

Specifically, the SBT’s assertion that close proximity to social resources is the baseline assumption of the human brain can be also conceptualized as follows: from an FEP perspective one could argue that at a relatively high level of the neurocognitive hierarchy, humans have the prediction that they need to maintain proximity with the caregiver. This prediction, or prior hypothesis in Bayesian terminology, is phylogenetically determined by the fact that humans are born to a long period of complete dependency on others for survival. This prior must also be ontogenetically determined in the sense that the particular history of such dependency and the parental and social environment each individual grows up into should inﬂuence the particular content of such priors. For example, as aforementioned and consistent with the tenets of attachment theory, someone who grew up with caring and available caregivers may be more likely to expect and to accept empathy and support from others than someone who was taken reared by unavailable or unreliable parents.

In the SBT it follows that self-regulation may require additional resources than social regulation, because in the case of the former the brain cannot rely on social resources for achieving required regulatory functions, e.g., protecting the organism from environmental threat. This assertion is entirely compatible with the predictions of the FEP, but it constitutes only one facet of the more complex social phenomena that this theory can explain. Specifically, from the point of view of the FEP, the need to self-regulate would indeed entail greater levels of prediction error that need to be minimized (free energy or surprise in the terminology of this theory) in lower hierarchical levels, because of the aforementioned fundamental (higher-level) expectation of social attachment and proximity. However, the FEP also presupposes a more general conﬂict between such social expectations and other self-serving motivations. For example, social proximity also requires competition for basic homeostatic resources and carries the risk of social aggression, attack, or intrusion (even by pro-social, affiliative activities such as birth or sex). Thus, at any given interaction the organism must balance the need for social proximity with the needs for homeostatic stability and bodily integrity. According to the FEP, the resolution of such conﬂicts relies on neuromodulatory (weighting, see above) functions and corresponding chemistries, e.g., neurotransmitters such as dopamine and neuropeptides such as oxytocin (Fotopoulou, 2013; Krahé et al., 2013; Quattrocki and Friston, 2014). In the case of labor pains, for example, the release of oxytocin seems to act to increase the precision of prosocial predictions about the importance of childbearing and thus reduce the precision of predictions errors regarding the painful and bodily threatening experience of birth (see also Krahé et al., 2013).

More generally, the FEP presupposes a basic conﬂict between perception and action that also applies to social phenomena. The organism could easily solve all discrepancies between prediction and error (e.g., hunger states) by simply changing its predictions (convincing oneself that one does not need food) and thus avoiding action (actively reducing prediction errors). This would of course cause homeostatic danger so the organism solves the conﬂict by attenuating (under-weighting) the ascending sensory prediction errors of self-generated actions to enable descending predictions to be fulfilled by peripheral and autonomic reﬂexes. This interpretation corresponds to the well-established phenomenon of sensory attenuation, the observation that the sensory consequences of self-generated actions are perceived as less intense. This phenomenon has been inﬂuentially illustrated by the experimental confirmation of the intuitive knowledge that one cannot tickle oneself (Weiskrantz et al., 1971). Interestingly, sensory attenuation is associated with the ability to discriminate between self and other. In the domain of empathy and social support, the distinction between self and other is almost as important as the sense of attachment and social connectedness (Decety and Lamm, 2007; Silani et al., 2013). For example, when an individual in pain receives active social support by their partner, or perceives them to be empathic, perceiving the partner themselves as being “worried”, or as needing to necessarily act “on behalf” of the sufferer (i.e., not differentiating sufficiently between self and other), has been noted to lead to increased perception of pain (Hurter et al., 2014), decreased feelings of self-efficacy and poor long-term clinical outcomes in the case of chronic pain (see Krahé et al., 2013 for review). In other terms, the FEP can explain why relying on other people’s resources may in certain situations lead to perceptual distortions (instead of appropriate self-protective actions) that are not beneficial to health.

In summary, although the two frameworks are highly compatible, the FEP to has a wider scope, accounting for both the positive and negative effects of social support and empathy.

## When predictions meet social expectations: placebo effects

The use of placebos in medicine, outside clinical trials, may be ethically and legally controversial, but is in fact quite frequent (Fässler et al., 2010). Results from a national survey of US internists and rheumatologists indicates that the prescriptions of placebo treatments such as vitamins and over the counter analgesics are commonly employed (Tilburt et al., 2008). Placebos are treatments whose benefits derive from positive patient expectations and not from the pharmacological component itself. Thus, placebo effects are a good example of how expectations, beliefs, or hope in patients is derived from the clinical relationship between the patient and her/his physician (Miller and Rosenstein, 2006). An impressive body of work during the past decade has elucidated the neurobiological mechanisms underlying placebo hypoalgesia (i.e., a decrease pain sensation). This hypoalgesia results from the activation of the opiodergic descending pain control system (Eippert et al., 2009). Another mechanism has also been proposed that relies on classical conditioning, consistent with a predictive coding perspective. In addition to their direct analgesic effects, opioids play a role in signaling top-down predictions in a generative model, and thus the ascending system is complemented by a descending system (Büchel et al., 2014). This descending system, amenable to conditioning (e.g., when individuals associate hospital with effective treatment and symptom reduction), originates in the rostral anterior cingulate cortex and anterior insula, and has reciprocal connections with the amygdala and periaqueductal gray (Wager, 2005). A study using functional molecular imaging in healthy participants found that activation of the nucleus accumbens dopamine release was observed during placebo administration and related to its anticipated effects, perception-anticipation mismatches, and placebo effect development (Scott et al., 2007). Furthermore, the magnitude of the placebo-induced dopaminergic response, measured during the expectation of pain, predicted the subsequent development of placebo-induced analgesia in pain trials.

While most placebo research has focused on understanding the underlying neurobiological mechanisms of the patient’s response to placebos, less effort has been directed to understanding the physician component of the clinical dyad. One notable functional MRI study explored brain responses when physicians interacted with their patients during a treatment or no-treatment conditions. Results showed that brain regions previously implicated in reward and subjective value (ventrolateral and dorsolateral prefrontal cortices and ventral striatum) were selectively activated during the treatment condition (Jensen et al., 2014). In addition, physician’s ability to take the patients’ perspective predicted neural response in the rostral anterior cingulate cortex, another region that has been associated with processing of reward.

Finally, this conceptual framework may also help explain why sometimes the best medicine is simply having someone to care for you, which seems to be at the core of many alternative medicines. Studies have shown that alternative treatments such as acupuncture tend to produce a larger placebo effect than, for example, handing out sugar pills, presumably because these alternative treatments involve more ritual, and thus raise patients’ expectations (Freedman, 2011).

## Conclusions

Throughout history, doctor-patient relationships have been acknowledged as having an important therapeutic effect, beyond the effects of prescribed drugs and other treatments (Di Blasi et al., 2001). Although numerous factors inﬂuence patients’ enablement, their perceptions of their doctors’ empathy and caring attitude are of key importance in contributing to patient outcomes in clinical practice. The beneficial impact of empathy on others can be explained by neurocognitive theories that emphasize the importance of social interactions, support, relations, and cognitions in health, as well as by neurocomputational theories which conceptualize the brain as an inferential, self-organizing machine which constantly makes predictions about the world and then optimizes them based on what it senses. Further empirical work at both behavioral and neurobiological levels is needed in order to test these theories in the context of clinical practice. However, their theoretical advantage lies in the fact that they suggest specific neurobiological mechanisms by which psychosocial variables such as interpersonal therapy can inﬂuence individual bodily perceptions and health outcomes. Moreover, both theories account for contextual and dispositional factors by which a clinician’s empathy may affect a patient’s beliefs and experiences and ultimately their health.

Thus, the benefit of physician’s empathy for a patient cannot be conceptualized as a mere dodo bird conjecture. Nor should clinical empathy be considered as a simple subjective humanistic stance. Furthermore, an empathetic attitude is not opposed to the objectivity of other medical skills and it can be studied with the scientific rigor applied to other biological domains. Specific expectations between the patient and her/his physician, when met and cared for, reduce uncertainty, and play a beneficial and crucial role in healing. A caring practitioner who takes more time to bond effectively with patients is an enormous boon to health. It benefits patients to have a doctor who spends more time with them and listens carefully. Many patients feel better simply when practitioners actively try to help them deal with vague, hard-to-diagnose complaints such as pain and fatigue, instead of telling them that there is no diagnosis or effective treatment. In psychotherapies, patients with positive attitudes who interact with a warm and genuine therapist (a factor known as the therapeutic alliance) have a better chance of experiencing clinical improvement, regardless of the therapist methods (Despland et al., 2009).

Empathy is just one of the elements that facilitates treatment effectiveness, but a powerful one.

## References

[B1] Anacker A. M. J. Beery A. K. (2013). Life in groups: the roles of oxytocin in mammalian sociality. Front. Behav. Neurosci. 7:185. 10.3389/fnbeh.2013.00185 24376404 PMC3858648

[B2] Beckes L. Coan J. A. (2011). Social baseline theory: the role of social proximity in emotion and economy of action. Soc. Personal. Psychol. Compass 5, 976–988 10.1111/j.1751-9004.2011.00400.x

[B4] Blatt B. LeLacheur S. F. Galinsky A. D. Simmens S. J. Greenberg L. (2010). Does perspective-taking increase patient satisfaction in medical encounters? Acad. Med. 85, 1445–1452. 10.1097/ACM.0b013e3181eae5ec 20736672

[B5] Brown E. C. Brüne M. (2012). The role of prediction in social neuroscience. Front. Hum. Neurosci. 6:147. 10.3389/fnhum.2012.00147 22654749 PMC3359591

[B6] Brown J. L. Sheffield D. Leary M. R. Robinson M. E. (2003). Social support and experimental pain. Psychosom. Med. 65, 276–283. 10.1097/01.psy.0000030388.62434.46 12651995

[B7] Büchel C. Geuter S. Sprenger C. Eippert F. (2014). Placebo analgesia: a predictive coding perspective. Neuron 81, 1223–1239. 10.1016/j.neuron.2014.02.042 24656247

[B8] Cacioppo J. T. Hawkley L. (2009). Perceived social isolation and cognition. Trends Cogn. Sci. 13, 447–454. 10.1016/j.tics.2009.06.005 19726219 PMC2752489

[B9] Cacioppo J. T. Hawkley L. C. Norman G. J. Berntson G. G. (2012). Social isolation. Ann. N Y Acad. Sci. 1231, 17–22 10.1111/j.1749-6632.2011.06028.x 21651565 PMC3166409

[B10] Cardoso C. Ellenbogen M. A. Serravalle L. Linnen A. M. (2013). Stress-induced negative mood moderates the relation between oxytocin administration and trust: evidence for the tend-and-befriend response to stress? Psychoneuroendocrinology 38, 2800–2804. 10.1016/j.psyneuen.2013.05.006 23768973

[B11] Cheng Y. Lin C. Liu H. L. Hsu Y. Lim K. Hung D.. (2007). Expertise modulates the perception of pain in others. Curr. Biol. 17, 1708–1713. 10.1016/j.cub.2007.09.020 17900903

[B12] Coan J. A. (2011). “The social regulation of emotion,” in The Oxford Handbook of Social Neuroscience, eds Decety J. Cacioppo J. T. (New York: Oxford University Press), 614–623.

[B13] Coan J. A. Kasle S. Jackson A. Schaefer H. S. Davidson R. J. (2013). Mutuality and the social regulation of neural threat responding. Attach. Hum. Dev. 15, 303–315. 10.1080/14616734.2013.782656 23547803 PMC4260393

[B14] Coan J. A. Maresh E. L. (2014). “Social baseline theory and the social regulation of emotion,” in The Handbook of Emotion Regulation, 2nd Edn., ed Gross J. (New York: The Guilford Press), 221–238.

[B15] Coan J. A. Schaefer H. S. Davidson R. J. (2006). Lending a hand: social regulation of the neural response to threat. Psychol. Sci. 17, 1032–1039. 10.1111/j.1467-9280.2006.01832.x 17201784

[B16] Collins N. L. Feeney B. C. (2004). Working models of attachment shape perceptions of social support: evidence from experimental and observational studies. J. Pers. Soc. Psychol. 87, 363–383. 10.1037/0022-3514.87.3.363 15382986

[B17] Coplan A. Goldie P. (2011). Empathy—Philosophical and Psychological Perspectives. New York: Oxford University Press.

[B18] Coutinho J. F. Silva P. Decety J. (2014). Neurosciences, empathy and healthy interpersonal relationships: recent findings and implications for counseling psychology. J. Couns. Psychol. 61, 541–548. 10.1037/cou0000021 25285714

[B19] Craig A. D. (2002). How do you feel? Interoception: the sense of the physiological condition of the body. Nat. Rev. Neurosci. 3, 655–666. 10.1038/nrn894 12154366

[B20] D’Amato F. N. Pavone F. (1993). Endogenous opioids: a proximate reward mechanism for kin selection? Behav. Neural Biol. 83, 79–83. 10.1016/0163-1047(93)90768-d 8216163

[B21] Davidov M. Zahn-Waxler C. Roth-Hanania R. Knafo A. (2013). Concern for others in the first year of life: theory, evidence and avenues for research. Child Dev. Perspect. 7, 126–131 10.1111/cdep.12028

[B22] Dayan P. Hinton G. E. Neal R. M. Zemel R. S. (1995). The Helmholtz machine. Neural Comput. 7, 889–904. 10.1162/neco.1995.7.5.889 7584891

[B24] Decety J. (2009). Empathy, sympathy and the perception of pain. Pain 145, 365–366. 10.1016/j.pain.2009.08.006 19716658

[B25] Decety J. (2011). Dissecting the neural mechanisms mediating empathy. Emot. Rev. 3, 92–108 10.1177/1754073910374662

[B26] Decety J. (2012). Empathy—From Bench to Bedside. Cambridge: MIT Press.

[B27] Decety J. Cowell J. M. (2014). Friends or foes: is empathy necessary for moral behavior? Perspect. Psychol. Sci. 9, 525–537. 10.1177/1745691614545130 25429304 PMC4241340

[B23] Decety J. Ickes W. (2009). The Social Neuroscience of Empathy. Cambridge: MIT Press.

[B28] Decety J. Jackson P. L. (2004). The functional architecture of human empathy. Behav. Cogn. Neurosci. Rev. 3, 71–100. 10.1177/1534582304267187 15537986

[B29] Decety J. Lamm C. (2007). The role of the right temporoparietal junction in social interaction: how low-level computational processes contribute to meta-cognition. Neuroscientist 13, 580–593. 10.1177/1073858407304654 17911216

[B30] Decety J. Meyer M. (2008). From emotion resonance to empathic understanding: a social developmental neuroscience account. Dev. Psychopathol. 20, 1053–1080. 10.1017/S0954579408000503 18838031

[B31] Decety J. Norman G. J. Berntson G. G. Cacioppo J. T. (2012). A neurobehavioral evolutionary perspective on the mechanisms underlying empathy. Prog. Neurobiol. 98, 38–48. 10.1016/j.pneurobio.2012.05.001 22580447

[B32] Decety J. Skelly L. (2014). “The neural underpinnings of the experience of empathy: lessons for psychopathy,” in The Oxford Handbook of Cognitive Neuroscience—Volume 2, eds Ochsner K. N. Kosslyn S. M. (New York: Oxford University Press), 228–243.

[B33] Decety J. Svetlova M. (2012). Putting together phylogenetic and ontogenetic perspectives on empathy. Dev. Cogn. Neurosci. 2, 1–24. 10.1016/j.dcn.2011.05.003 22682726 PMC6987713

[B34] Decety J. Yang C. Y. Cheng Y. (2010). Physicians down regulate their pain empathy response: an event-related brain potential study. Neuroimage 50, 1676–1682. 10.1016/j.neuroimage.2010.01.025 20080194

[B35] Derntl B. Finkelmeyer A. Eickoff S. Kellerman T. Falkenberg D. I. Schneider F.. (2010). Multidimensional assessment of empathic abilities: neural correlates and gender differences. Psychoneuroendocrinology 25, 67–82. 10.1016/j.psyneuen.2009.10.006 19914001

[B36] Despland J.-N. de Roten Y. Drapeau M. Currat T. Beretta V. Kramer U. (2009). The role of alliance in the relationship between therapist competence and outcome in brief psychodynamic psychotherapy. J. Nerv. Ment. Dis. 197, 362–367. 10.1097/NMD.0b013e3181a20849 19440110

[B3] Di Blasi Z. Harkness E. Ernst E. Georgiou A. Kleijnen J. (2001). Inﬂuence of context effects on health outcomes: a systematic review. Lancet 357, 757–762. 10.1016/s0140-6736(00)04169-6 11253970

[B37] Ditzen B. Schmidt S. Strauss B. Nater U. M. Ehlert U. Heinrichs M. (2008). Adult attachment and social support interact to reduce psychological but not cortisol responses to stress. J. Psychosom. Res. 64, 479–486. 10.1016/j.jpsychores.2007.11.011 18440400

[B38] Domes G. Sibold M. Schulze L. Lischke A. Herpertz S. C. Heinrichs M. (2013). Intranasal oxytocin increases covert attention to positive social cues. Psychol. Med. 43, 1747–1753. 10.1017/S0033291712002565 23146328

[B39] Eippert F. Bingel U. Schoell E. D. Yacubian J. Klinger R. Lorenz J.. (2009). Activation of the opiodergic descending pain control system underlies placebo analgesia. Neuron 63, 533–543. 10.1016/j.neuron.2009.07.014 19709634

[B40] Eisenberger N. I. Taylor S. E. Gable S. L. Hilmert C. J. Lieberman M. D. (2007). Neural pathways link social support to attenuated neuroendocrine stress responses. Neuroimage 35, 1601–1612. 10.1016/j.neuroimage.2007.01.038 17395493 PMC2710966

[B41] Farrow T. Woodruff P. (2007). Empathy in Mental Illness. Cambridge: Cambridge University Press.

[B42] Fässler M. Meissner K. Scnedider A. Linde K. (2010). Frequency and circumstances of placebo use in clinical practice - a systematic review of empirical studies. BMC Med. 8:15. 10.1186/1741-7015-8-15 20178561 PMC2837612

[B43] Figley C. R. (2012). “The empathic response in clinical practive: antecedents and consequences,” in Empathy—From Bench to Bedside, ed Decety J. (Cambridge: MIT Press), 263–273.

[B44] Fotopoulou A. (2013). Beyond the reward principle: consciousness as precision seeking. Neuropsychoanalysis 15, 33–38 10.1080/15294145.2013.10773715

[B45] Freedman D. H. (2011). The Triumph of new-age medicine. Atlantic 308, 90–100 10.1037/e581592011-002

[B46] Friston K. J. (2005). A theory of cortical responses. Philos. Trans. R. Soc. Lond. B Biol. Sci. 360, 815–836. 10.1098/rstb.2005.1622 15937014 PMC1569488

[B47] Friston K. J. (2010). The free-energy principle: a unified brain theory? Nat. Rev. Neurosci. 11, 127–138. 10.1038/nrn2787 20068583

[B48] Gleichgerrcht E. Decety J. (2012). “The costs of empathy among health professionals,” in Empathy: From Bench to Bedside, ed Decety J. (Cambridge: MIT Press), 245–261.

[B49] Gleichgerrcht E. Decety J. (2013). Empathy in clinical practice: how individual dispositions, gender and experience moderate empathic concern, burnout and emotional distress in physicians. PLoS One 8:e61526. 10.1371/journal.pone.0061526 23620760 PMC3631218

[B50] Gleichgerrcht E. Decety J. (2014). The relationship between different facets of empathy, pain perception and compassion fatigue among physicians. Front. Behav. Neurosci. 8:243. 10.3389/fnbeh.2014.00243 25071495 PMC4093939

[B51] Goubert L. Craig K. D. Buysse A. (2009). “Perceiving others in pain: experimental and clinical evidence on the role of empathy,” in The Social Neuroscience of Empathy, eds Decety J. Ickles W. (Cambridge: MIT Press), 153–165.

[B52] Graustella A. J. MacLeod C. (2012). A critical review of the inﬂuence of oxytocin nasal spray on social cognition in humans: evidence and future directions. Horm. Behav. 61, 410–418. 10.1016/j.yhbeh.2012.01.002 22265852

[B53] Gross E. B. Proffitt D. (2013). The economy of social resources and its inﬂuence on spatial perceptions. Front. Hum. Neurosci. 7:772. 10.3389/fnhum.2013.00772 24312039 PMC3832788

[B54] Gunnar M. Quevedo K. (2007). The neurobiology of stress and development. Annu. Rev. Psychol. 58, 145–173. 10.1146/annurev.psych.58.110405.085605 16903808

[B55] Halpern J. (2012). “Clinical empathy in medical care,” in Empathy: From Bench to Bedside, ed Decety J. (Cambridge: MIT Press), 229–244.

[B56] Hennessy M. B. Kaiser S. Sachser N. (2009). Social buffering of the stress response: diversity, mechanisms and functions. Front. Neuroendocrinol. 30, 470–482. 10.1016/j.yfrne.2009.06.001 19545584

[B57] Hinton G. E. van Camp D. (1993). Keeping neural networks simple by minimizing the description length of weights. Proc. COLT 5–13 10.1145/168304.168306

[B58] Hirsch E. M. (2007). Virtual mentor. Am. Med. Assoc. J. Ethics 9, 423–427. 10.1001/virtualmentor.2007.9.6.medu1-0706 23218048

[B59] Ho S. S. Konrath S. Brown S. Swain J. E. (2014). Empathy and stress related neural responses in maternal decision making. Front. Neurosci. 8:152. 10.3389/fnins.2014.00152 24971049 PMC4053926

[B60] Hojat M. Louis D. Z. Markham F. W. Wender R. Rabinowitz C. Gonnella J. S. (2011). Physicians’ empathy and clinical outcomes for diabetic patients. Acad. Med. 86, 359–364. 10.1097/ACM.0b013e3182086fe1 21248604

[B61] Hostinar C. E. Stellern S. A. Schaefer C. Carlson S. M. Gunnar M. R. (2012). Asssociations between early life adversity and executive function in children adopted internationally from orphanages. Proc. Natl. Acad. Sci. U S A 109, 17208–17212. 10.1073/pnas.1121246109 23047689 PMC3477377

[B62] Hostinar C. E. Sullivan R. M. Gunnar M. R. (2014). Psychobiological mechanisms underlying the social buffering of the hypothalamic-pituitary-adrenocortical axis: a review of animal models and human studies across development. Psychol. Bull. 140, 256–282. 10.1037/a0032671 23607429 PMC3844011

[B63] Huntington B. Kuhn N. (2003). Communication gaffes: a root cause of malpractice claims. Proc. (Bayl. Univ. Med. Cent.) 75201, 157–161. 16278732 10.1080/08998280.2003.11927898 PMC1201002

[B64] Hurter S. Paloyelis Y. de C. Williams A. C. Fotopoulou A. (2014). Partners’ empathy increases pain ratings: effects of perceived empathy and attachment style on pain report and display. J. Pain 15, 934–944. 10.1016/j.jpain.2014.06.004 24953886 PMC4162650

[B65] Jackson T. Huang X. Chen H. Phillips H. (2009). Effects of threatening information on interpersonal responses to pain. Eur. J. Pain 13, 431–438. 10.1016/j.ejpain.2008.05.012 18602319

[B66] Jackson T. Iezzi T. Chen H. Ebnet S. Eglitis K. (2005). Gender, interpersonal transactions and the perception of pain: an experimental analysis. J. Pain 6, 228–236. 10.1016/j.jpain.2004.12.004 15820910

[B67] Jensen K. B. Petrovic P. Kerr C. E. Kirsch I. Raicek J. Cheetham A.. (2014). Sharing pain and relief: neural correlates of physicians during treatment of patients. Mol. Psychiatry 19, 392–398. 10.1038/mp.2012.195 23358155 PMC3981541

[B68] Kelley J. M. Kraft-Todd G. Schapira L. Kossowsky J. Riess H. (2014). The inﬂuence of the patient-clinician relationship on healthcare outcomes: a systematic review and meta-analysis of randomized controlled trials. PLoS One 9:e94207. 10.1371/journal.pone.0094207 24718585 PMC3981763

[B69] Kestenbaum R. Farber E. A. Sroufe L. A. (1989). Individual differences in empathy among preschoolers: relation to attachment history. New Dir. Child Dev. 44, 51–64. 10.1002/cd.23219894405 2771129

[B71] Koster-Hale J. Saxe R. (2013). Theory of mind: a neural prediction problem. Neuron 79, 836–848. 10.1016/j.neuron.2013.08.020 24012000 PMC4041537

[B72] Krahé C. Springer A. Weinman J. A. Fotopoulou A. (2013). The social modulation of pain: others as predictive signals of salience - a systematic review. Front. Hum. Neurosci. 7:386. 10.3389/fnhum.2013.00386 23888136 PMC3719078

[B73] Krasner M. S. Epstein R. M. Beckman H. Suchman A. L. Chapman B. Mooney C. J.. (2009). Association of an educational program in mindful communication with burnout, empathy and atttitudes among primary care physicians. JAMA 302, 1284–1293. 10.1001/jama.2009.1384 19773563

[B74] Kumsta R. Heinrichs M. (2013). Oxytocin, stress and social behavior: neurogenetics of the human oxytocin system. Curr. Opin. Neurobiol. 23, 11–16. 10.1016/j.conb.2012.09.004 23040540

[B75] Langford D. J. Tuttle A. H. Brown K. Deschenes S. Fischer D. B. Mutso A.. (2010). Social approach to pain in laboratory mice. Soc. Neurosci. 5, 163–170. 10.1080/17470910903216609 19844845

[B76] Lawson R. P. Rees G. Friston K. J. (2014). An aberrant precision account of autism. Front. Hum. Neurosci. 8:302. 10.3389/fnhum.2014.00302 24860482 PMC4030191

[B78] Mallinckrodt B. (2000). Attachment, social competencies, social support and interpersonal process in psychotherapy. Psychother. Res. 10, 239–266 10.1093/ptr/10.3.239

[B79] Master S. L. Eisenberger N. I. Taylor S. E. Naliboff B. D. Shirinyan D. Lieberman M. D. (2009). A picture’s worth: partner photographs reduce experimentally induced pain. Psychol. Sci. 20, 1316–1318. 10.1111/j.1467-9280.2009.02444.x 19788531

[B81] Mercer S. W. Jani B. D. Maxwell M. Wong S. Y. S. Watt G. C. M. (2012). Patient enablement requires physician empathy: a cross-sectional study of general practice consultations in areas of high and low socioeconomic deprivation in Scotland. BMC Fam. Pract. 13:6. 10.1186/1471-2296-13-6 22316293 PMC3329411

[B80] Mercer S. W. Reynolds W. J. (2002). Empathy and quality of care. Br. J. Gen. Pract. 52, S9–S12. 12389763 PMC1316134

[B82] Meredith P. Ownsworth T. Strong J. (2008). A review of the evidence linking adult attachment theory and chronic pain: presenting a conceptual model. Clin. Psychol. Rev. 28, 407–429. 10.1016/j.cpr.2007.07.009 17719157

[B83] Mikulincer M. Gillath O. Halevy V. Avihou N. Avidan S. Eshkoli N. (2001). Attachment theory and reactions to others’ needs: evidence that activation of the sense of attachment security promotes empathic responses. J. Pers. Soc. Psychol. 81, 1205–1224. 10.1037/0022-3514.81.6.1205 11761318

[B84] Miller F. G. Rosenstein D. L. (2006). The nature and power of the placebo effect. J. Clin. Epidemiol. 59, 331–335. 10.1016/j.jclinepi.2005.12.001 16549251

[B85] Montague E. Chen P. Xu J. Chewning B. A. Barrett B. (2013). Nonverbal interpersonal interactions in clinical encounters and patient perceptions of empathy. J. Particip. Med. 5:e33.

[B86] Montoya P. Larbig W. Braun C. Preissl H. Birbaumer N. (2004). Inﬂuence of social support and emotional context on pain processing and magnetic brain responses in fibromyalgia. Arthritis Rheum. 50, 4035–4044. 10.1002/art.20660 15593181

[B87] Norman G. J. Cacioppo J. T. Morris J. S. Malarkey W. B. Berntson G. G. Devries A. C. (2011). Oxytocin increases autonomic cardiac control: moderation by loneliness. Biol. Psychol. 86, 174–180. 10.1016/j.biopsycho.2010.11.006 21126557

[B88] Ozbay F. Johnson D. C. Dimoulas E. Morgan C. A. Charney D. Southwick S. (2007). Social support and resilience to stress. Psychiatry 4, 35–45. 20806028 PMC2921311

[B89] Paloyelis Y. Doyle O. M. Zelaya F. O. Maltezos F. Williams S. C. Fotopoulou A. (2014). A spatiotemporal profile of in vivo cerebral blood ﬂow changes following intranasal oxytocin in humans. Biol. Psychiatry [Epub ahead of print]. 10.1016/j.biopsych.2014.10.005 25499958

[B90] Pedersen R. (2010). Empathy development in medical education - a critical review. Med. Teach. 32, 593–600. 10.3109/01421590903544702 20653383

[B91] Pellicano E. Burr D. (2012). When the world becomes “too real”: a Bayesian explanation of autistic perception. Trends Cogn. Sci. 16, 504–510. 10.1016/j.tics.2012.08.009 22959875

[B92] Quattrocki E. Friston K. (2014). Autism, oxytocin and interoception. Neurosci. Biobehav. Rev. [Epub ahead of print]. 30, 410–430. 10.1016/j.neubiorev.2014.09.012 25277283 PMC4726659

[B93] Rakel D. P. Hoeft T. J. Barrett B. P. Chewning B. A. Craig B. M. Niu M. (2009). Practitioner empathy and the duration of the common cold. Fam. Med. 41, 494–501. 19582635 PMC2720820

[B94] Reblin M. Uchino B. N. (2009). Social and emotional support and its implication for health. Curr. Opin. Psychiatry 21, 201–205. 10.1097/YCO.0b013e3282f3ad89 18332671 PMC2729718

[B95] Riess H. Kelley J. M. Bailey R. W. Dunn E. J. Phillips M. (2012). Empathy training for resident physicians: a randomized controlled trial of a neuroscience-informed curriculum. J. Gen. Intern. Med. 27, 1280–1286. 10.1007/s11606-012-2063-z 22549298 PMC3445669

[B96] Rodrigues S. M. Saslow L. R. Garcia N. John O. P. Keltner D. (2009). Oxytocin receptor genetic variation relates to empathy and stress reactivity in humans. Proc. Natl. Acad. Sci. U S A 106, 21437–21441. 10.1073/pnas.0909579106 19934046 PMC2795557

[B97] Sambo C. F. Howard M. Kopelman M. Williams S. Fotopoulou A. (2010). Knowing you care: effects of perceived empathy and attachment style on pain perception. Pain 151, 687–693. 10.1016/j.pain.2010.08.035 20869808

[B98] Scott D. J. Stohler C. S. Egnatuk C. M. Wang H. Koeppe R. A. Zubieta J.-K. (2007). Individual differences in reward responding explain placebo-induced expectations and effects. Neuron 55, 325–336. 10.1016/j.neuron.2007.06.028 17640532

[B99] Shamay-Tsoory S. G. Abu-Akel A. Palgi S. Sulieman R. Fischer-Shofty M. Levkovitz Y.. (2013). Giving peace a chance: oxytocin increases empathy to pain in the context of the Israeli-Palestinian conﬂict. Psychoneuroendocrinology 38, 3139–3144. 10.1016/j.psyneuen.2013.09.015 24099859

[B100] Shapiro J. (2012). “The paradox of teaching empathy in medical education,” in Empathy-From Bench to Bedside, ed Decety J. (Cambridge: MIT Press), 275–290.

[B101] Silani G. Lamm C. Ruff C. C. Singer T. (2013). Right supramarginal gyrus is crucial to overcome emotional egocentricity bias in social judgments. J. Neurosci. 33, 15466–15476. 10.1523/jneurosci.1488-13.2013 24068815 PMC6618458

[B102] Singer T. Lamm C. (2009). The social neuroscience of empathy. Ann. N Y Acad. Sci. 1156, 81–96. 10.1111/j.1749-6632.2009.04418.x 19338504

[B103] Smajdor A. Stöckl A. Salter C. (2011). The limits of empathy: problems in medical education and practice. J. Med. Ethics 37, 380–383. 10.1136/jme.2010.039628 21292696

[B104] Smith K. E. Porges E. C. Norman G. J. Connelly J. J. Decety J. (2014). Oxytocin receptor (OXTR) gene variation predicts empathic concern and autonomic arousal while perceiving harm to others. Soc. Neurosci. 9, 1–9. 10.1080/17470919.2013.863223 24295535 PMC3923324

[B105] Taylor S. E. (2007). “Social support,” in Foundations of Health Psychology, eds Silver H. Friedman R. C. (New York: Oxford University Press), 145–171.

[B106] Tilburt J. C. Emanuel E. J. Kaptchuk T. J. Curlin F. A. Miller F. G. (2008). Prescribing placebo treatments: results of national survey of US internists and rheumatologists. BMJ 337:a1938. 10.1136/bmj.a1938 18948346 PMC2572204

[B107] Uchino B. N. Cacioppo J. T. Kiecolt-Glaser J. K. (1996). The relationship between social support and physiological processes: a review with emphasis on underlying mechanisms and implications for health. Psychol. Bull. 119, 488–531. 10.1037/0033-2909.119.3.488 8668748

[B108] Wager T. D. (2005). The neural bases of placebo effects in pain. Curr. Dir. Psychol. Sci. 14, 175–179 10.1016/j.spmd.2005.02.003

[B109] Weinfield N. S. Srouffe L. A. Egeland B. Carlson E. (2010). “Individual differences in infant-caregiver attachment,” in Handbook of Attachment, eds Cassidy J. Shaver P. R. (New York: The Guilford Press), 78–101.

[B110] Weiskrantz L. Elliott J. Darlington C. (1971). Preliminary observations on tickling oneself. Nature 230, 598–599. 10.1038/230598a0 4928671

[B111] Wiech K. Lin C. Brodersen K. H. Bingel U. Ploner M. Tracey I. (2010). Anterior insula integrates information about salience into perceptual decisions about pain. J. Neurosci. 30, 16324–16331. 10.1523/JNEUROSCI.2087-10.2010 21123578 PMC6634837

[B112] Williams A. O’Driscoll K. Moore C. (2014). The inﬂuence of empathic concern on prosocial behavior in children. Front. Psychol. 5:425. 10.3389/fpsyg.2014.00425 24860537 PMC4026684

[B113] Younger J. Aron A. Parke S. Chatterjee N. Mackey S. C. (2010). Viewing pictures of a romantic partner reduces experimental pain: involvement of neural reward systems. PLoS One 5:e13309. 10.1371/journal.pone.0013309 20967200 PMC2954158

